# Thrift and Health

**Published:** 1860-04

**Authors:** 


### THRIFT AND HEALTH.

By returns made to the Registrar-General in France, it appears that persons who are "well to do" live, on an average, eleven years longer than those who are dependent on their daily labor. One reason for this is, the health-giving influence of composure of mind; another, that forehandedness removes the necessity for hard exposures. The same important truth is shown by the fact that the average life of those who belong to the Society of Friends in England is some fifteen years greater than of others in the same sphere of life, the Friends being, the world over, models of thrift and quiet composure.

As judicious economy promotes thrift, we propose it to our readers as a good medicine—a medicine safe and efficient, applicable to all climes, countries, and classes. It is "hard to take" to some, but steady persistence in its practice soon makes it a habit, when it is rather easier to be economical than to be extravagant.

Extravagance, waste, and carelessness not only ruin those who practice them, but have a demoralizing effect on those who may be benefited thereby in a material point of view. Persons seldom thrive whose occupations or modes of obtaining a living depend on chance, are in a great measure fortuitous or uncertain—such as gamblers, stock-brokers, robbers, wreckers, hunters, miners, office-holders, and speculators in general. Hence those parents are wisest who bring up their children to the expectation of making a living or of becoming rich by some one occupation which brings with it gains which are moderate, uniform, and steady. As a general rule to young men, the first political or salaried office, the first bet won, the first successful speculation, is at the same time the first step towards material unthrift, towards moral degradation, and towards a premature grave.

